# Signature of m5C-Related lncRNA for Prognostic Prediction and Immune Responses in Pancreatic Cancer

**DOI:** 10.1155/2022/7467797

**Published:** 2022-02-15

**Authors:** Xiangrong Liu, Dan Wang, Shiyu Han, Fang Wang, Jinfeng Zang, Caifeng Xu, Xue Dong

**Affiliations:** ^1^College of Nursing, Changchun University of Chinese Medicine, Changchun, China; ^2^Department of Endocrinology, Metabolism and Gastroenterology, The Third Affiliated Clinical Hospital of Changchun University of Chinese Medicine, Changchun, China; ^3^Department of Emergency, Qianwei Hospital of Jilin Province, Changchun, China; ^4^Department of Obstetrics & Gynecology, Army Military Medical University First Affiliated Hospital, Chongqing, China

## Abstract

**Background:**

Pancreatic cancer (PC) has a high mortality and dismal prognosis, predicting to be the second most lethal malignancy. 5-Methylcytosine (m5C) and long noncoding RNAs (lncRNAs) are both crucial in the prognostic outcome and immunotherapeutic effect for PC patients. Therefore, we aimed to create an m5C-related lncRNA signature (m5C-LS) for PC patients' prognosis and treatment.

**Methods:**

Clinicopathological information and RNAseq data were acquired from The Cancer Genome Atlas (TCGA) database. Pearson's correlation analysis was used to extract m5C-related lncRNAs in PC. Univariate, least absolute shrinkage and selection operator (LASSO), and multivariate Cox analyses were adopted to build an m5C-LS. Kaplan–Meier (K-M), principal component analysis (PCA), and nomogram were utilized to assess model accuracy. In addition, we explored the model's possible immunotherapeutic responses and drug sensitivity targets.

**Results:**

Three m5C-related lncRNAs were finally established to construct the risk signature, which has a good and independent predictive ability for PC patients. Based on the m5C-LS, patients were classified into the low- and high-m5C-LS group, with the latter having a worse prognosis. Furthermore, the m5C-LS allowed us to better discriminate the immunotherapeutic responses of PC patients in different subgroups.

**Conclusions:**

Our study constructed an m5C-LS and established a nomogram model that accurately predicted the prognosis of PC patients, as well as provides promising immunotherapeutic strategies in the future.

## 1. Introduction

Pancreatic cancer (PC) is a lethal disease with high mortality, having overtaken breast cancer to become the third top cause of cancer death in the United States in early 2017, which is expected to be the second cause by 2030 [1, 2]. According to the latest epidemiological data, 56,770 new instances of PC were discovered in the United States, while 45,750 patients died from the condition. It has a dismal prognosis as the majority of PC patients diagnosed at an advanced stage, with only a 9% five-year survival rate [3]. PC patients are staged from I to IV using the AJCC TNM staging criteria, and CT scan imaging was clinically used to group them into grades I–IV. Surgery is now the only possible way to cure PC and increase the 5-year survival rate to 20–30%. However, when a tumor is discovered, it is often already metastatic and spread, making surgical removal extremely difficult [4]. Despite advances in adjuvant treatment methods such as radiation, chemotherapy, and molecular targeted therapy, PC patients' survival rate remains dismal [5]. Therefore, finding new molecular biomarkers and therapeutic targets helps for the improvement of prognosis in PC.

It has been reported that RNA modification is critical for posttranscriptional gene expression regulation in various cancers [6]. There are over 100 distinct forms of RNA modification, including mRNA, microRNA, and long noncoding RNA (lncRNA) [7, 8]. Especially, lncRNAs are derived from noncoding sections of the genome that exceed 200 nucleotides in length [9]. Additionally, 5-methylcytosine (m5C) is a frequent methylation modification that plays a vital function in RNA metabolism such as RNA stability, export, recognition, and translation [10, 11]. The m5C sites have been confirmed to be abundantly distributed in lncRNA [12], but our understanding of how m5C is regulated in lncRNA is currently restricted. Therefore, investigating the role of m5C-related lncRNAs in the PC progression might be beneficial for finding prognostic targeting.

In this study, we extracted the expression patterns of 243 lncRNAs and 13 m5C genes from The Cancer Genome Atlas (TCGA) database. Pearson's correlation was then used to identify lncRNAs that were associated with m5C. A novel m5C-related lncRNA signature (m5C-LS) was finally constructed, which accurately predicted PC patients' OS. A nomogram integrating clinical features and this model was also established. Significantly, we identified prospective medicines targeting the m5C-LS, thereby providing direction for the therapy of PC.

## 2. Materials and Methods

### 2.1. Data Acquisition

TCGA (https://cancergenome.nih.gov/) database was used to retrieve the RNA transcriptome data, pertinent clinical information, and mutation data of PC patients. We collected a list of 13 m5C genes based on the existing research [13, 14]. Pearson's correlation analysis was implemented to screen for m5C-associated lncRNAs, and we found 243 m5C-related lncRNAs. The correlation coefficient |*R*| >0.4 and *p* <0.001 were utilized as criteria for the procedure.

### 2.2. Construction of the Predictive Signature

The complete TCGA dataset was randomly assigned to two subsets: a discovery and a testing cohort. The baseline features of these two groups are shown in Table S1. In the discovery cohort, we determined prognostic m5C-lncRNAs using univariate analysis (*p* < 0.05). Then, we discovered that four m5C-associated lncRNAs were differentially connected to the outcome of PC cases by the least absolute shrinkage and selection operator (LASSO) method. The four m5C-related lncRNAs were analyzed using multifactor Cox regression, and an m5C-LS was eventually developed. The risk factor of m5C-LS =  ∑exp(m5C − lncRNAs)*∗β*. *β* is the coefficient of each m5C-lncRNA from Cox analysis. Subgroups were created based on the median risk score, including low- and high-risk groups.

### 2.3. Gene Ontology (GO) Analysis

GO method was applied to find the possible biological function [15]. The R package clusterProfiler was used in this procedure [16]. The *p* value was used to define the analysis threshold, and *p* value <0.05 showed that the functional pathway was significantly enriched.

### 2.4. Immunotherapeutic Response Prediction

To analyze the mutation data, we utilized the R program maftools. The tumor-specific mutant genes were used to calculate the tumor mutational burden (TMB). We employed the TIDE algorithm to estimate the probability of an immunotherapeutic response.

### 2.5. Principal Component Analysis (PCA)

The whole-genome expression profiles, 13 m5C genes, three m5C-lncRNAs, and the m5C-LS were all analyzed using PCA [17] to achieve model identification. Kaplan–Meier (K-M) survival method was implemented to determine differences in clinical outcomes between the two groups.

### 2.6. Chemotherapy Response Prediction

To detect the ability of the m5C-LS, we assessed the half-maximal inhibitory concentration (IC_50_) to mirror the chemotherapeutic drug response. Using the R package pRRophetic [18], IC_50_ of drugs according to the Genomics of Drug Sensitivity in Cancer (GDSC) online tool was predicted for PC samples.

### 2.7. Independence of the m5C-LS

When additional clinical characteristics (gender, age, stage, and grade) were taken into consideration, the predictive pattern was assessed using multivariate and univariate Cox regression analyses in patients with PC to determine whether it was an independent predictor.

### 2.8. Establishing a Predictive Nomogram

The m5C-LS and other factors (age, gender, risk score, stage, and grade) were used to establish a predictive nomogram. Moreover, Hosmer–Lemeshow test was utilized to detect the exactness of the nomogram.

## 3. Results

### 3.1. Identification of the m5C-Related lncRNAs

**Figure 1:**
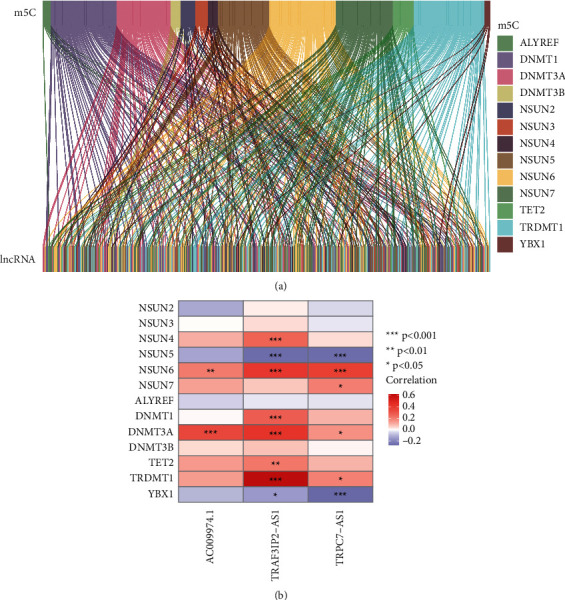
Selection of m5C-related lncRNAs in PC patients. (a) Sankey diagram for the network of m5C genes and related lncRNAs. (b) Heatmap for relationships between 13 m5C genes and 3 m5C-related lncRNAs. ^*∗*^*p* < 0.05, ^*∗∗*^*p* < 0.01, and ^*∗∗∗*^*p* < 0.001.

A total of 13 m5C genes and 14,056 lncRNAs were extracted from the PC dataset. m5C-related lncRNAs were defined as those with a significant link (*r* > 0.4 and *p* < 0.001) to one of the 13 m5C genes. Finally, the m5C-lncRNA coexpression network is shown in Figure 1(a). Throughout TCGA dataset, Figure 1(b) depicts the association between 13 m5C genes and three prognostic m5C-related lncRNAs.

### 3.2. Determination of the m5C-LS

**Figure 2:**
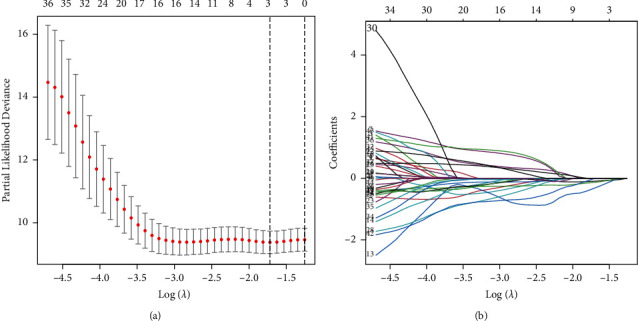
Description of m5C-LS. (a) The LASSO analysis of PC. (b) Determine the optimal LASSO settings.

Using univariate Cox regression analysis, we selected m5C-associated prognostic lncRNAs from 243 m5C-lncRNAs in the discovery cohort. In TCGA dataset, 45 m5C-related lncRNAs were substantially linked with OS (Table S2). A typical approach of multiple regression analysis, LASSO-penalized Cox, not only improves the statistical model's prediction accuracy but also allows for variable choices and regularization at the same time. We used LASSO analysis to reduce the overfitting of the m5C-LS, resulting in 45 m5C-lncRNAs remaining (Figures 2(a) and 2(b)). Finally, three m5C-related lncRNAs were screened in the discovery queue to create the m5C-LS for PC patients (Table 1).

**Figure 3:**
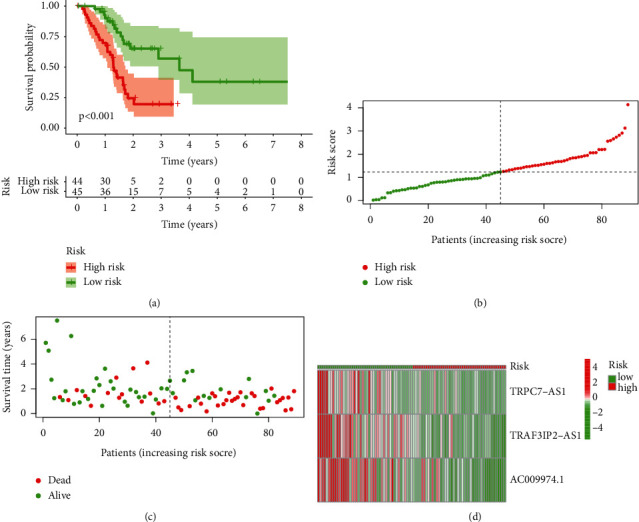
Identification of the prognostic value of m5C-LS in TCGA training set. (a) K-M curves of patients' OS between high- and low-m5C-LS groups. (b) Distribution of the risk score and patients. (c) Dot plot of survival status. (d) Heatmap of 3 m5C-related lncRNAs' expression between two groups.

Based on the median value of the prognostic risk grade, PC samples were divided into low- and high-risk groups. K-M analysis revealed a notable difference between two groups (*p* < 0.001, Figure 3(a)). The distribution of risk grades, survival status of cases, and expression of model lncRNAs are shown in Figures 3(b)–3(d).

**Figure 4:**
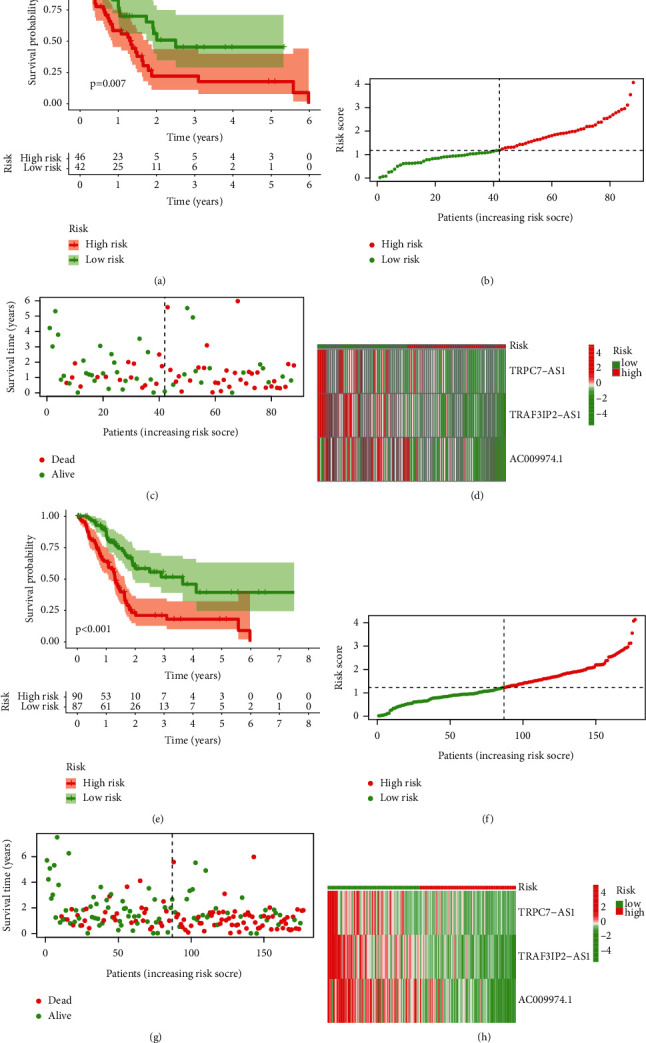
Verification of m5C-LS in TCGA testing and entire sets. (a, e) K-M curves of patients' OS between high- and low-m5C-LS groups. (b, f) Distribution of the risk score and patients. (c, g) Dot plot of survival status. (d, h) Heatmap of 3 m5C-related lncRNAs' expression between two risk groups.

We used the standard method to confirm the reliability of the m5C-LS. As we expected, a similar trend is verified in the verification cohorts (Figure 4).

**Figure 5:**
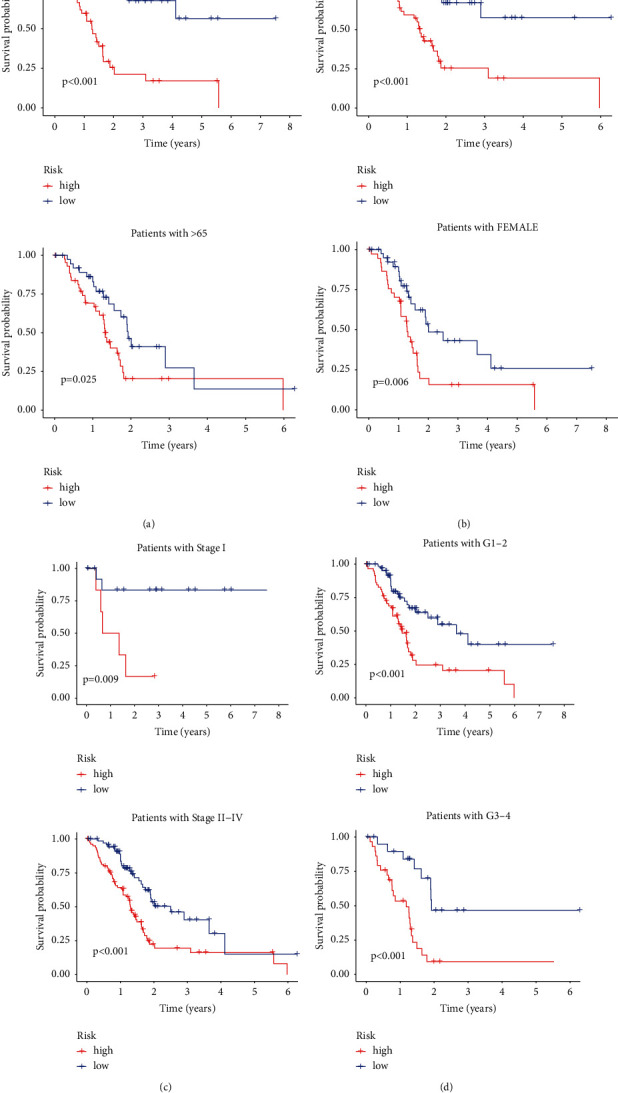
K-M curves of patients' OS grouped by (a) age, (b) gender, (c) TNM stage, and (d) tumor grade between two groups in TCGA entire set.

In TCGA-PC dataset, the disparities in the clinical outcome stratified by clinical features were studied between two groups. The patient outcome of the low-m5C-LS group remained superior to the high-m5C-LS, regardless of subgroups defined by gender, age, stage, or grade (Figure 5).

### 3.3. PCA of the m5C-LS

**Figure 6:**
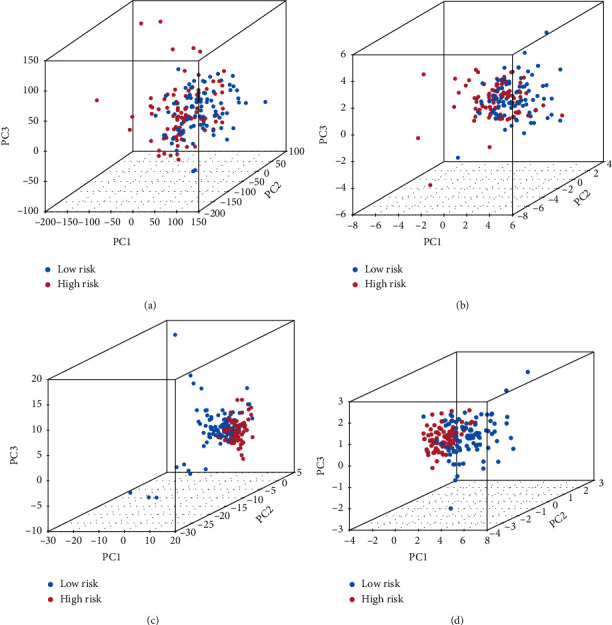
PCA comparison between two groups based on (a) entire gene profiles, (b) m5C coding genes, (c) m5C-related lncRNAs, and (d) m5C-LS in TCGA entire set.

PCA was used to examine the difference between the two risk groups. The distributions of the two groups were rather dispersed (Figure 6). These findings implied that the m5C-LS may differentiate between the two groups.

### 3.4. Clinical Value of the Signature

**Figure 7:**
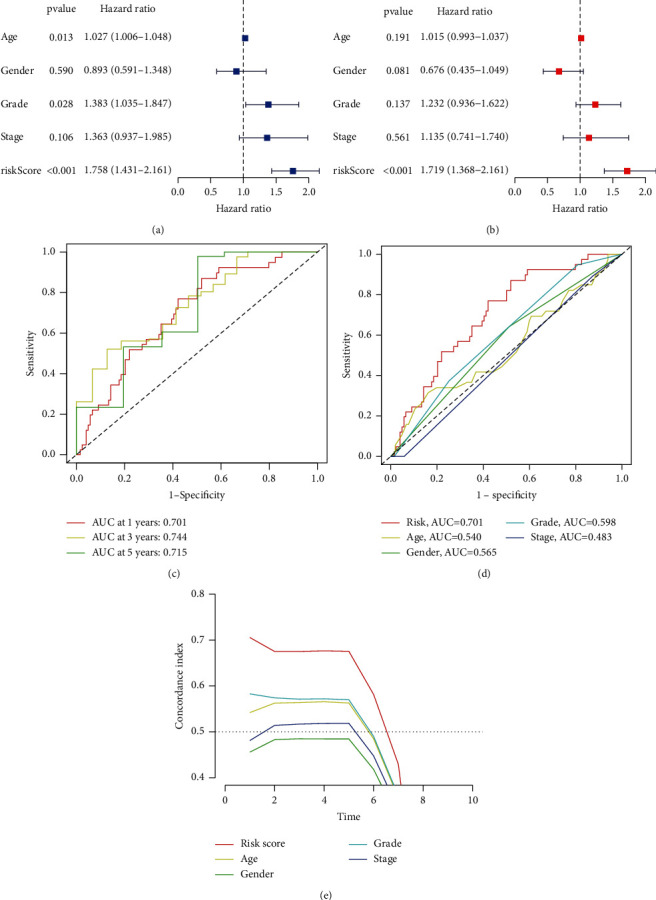
Evaluation of the m5C-LS prognostic value in TCGA entire set. (a, b) Univariate and multivariate analyses of PC patients' OS. (c) ROC curves of 1-, 3-, and 5-year survival. (d) ROC curves of both clinical features and risk score. (e) Concordance indexes of both clinical features and risk score.

Both univariate and multivariate methods unearthed the robust independence of our proposed m5C-LS (*p* < 0.001, Figures 7(a) and 7(b)).  Figure 7(c) shows that the AUC values for one, three, and five years are all more than 0.70, showing that this model had a high predictive value. The AUC of the risk grade was similarly greater than the AUCs of other clinical parameters, suggesting that the m5C-LS for PC was rather reliable (Figure 7(d)). The risk score's concordance index was usually higher than that of other clinical indicators as time went on, indicating the favorable performance of the m5C-LS (Figure 7(e)).

### 3.5. Construction of a Nomogram Model

**Figure 8:**
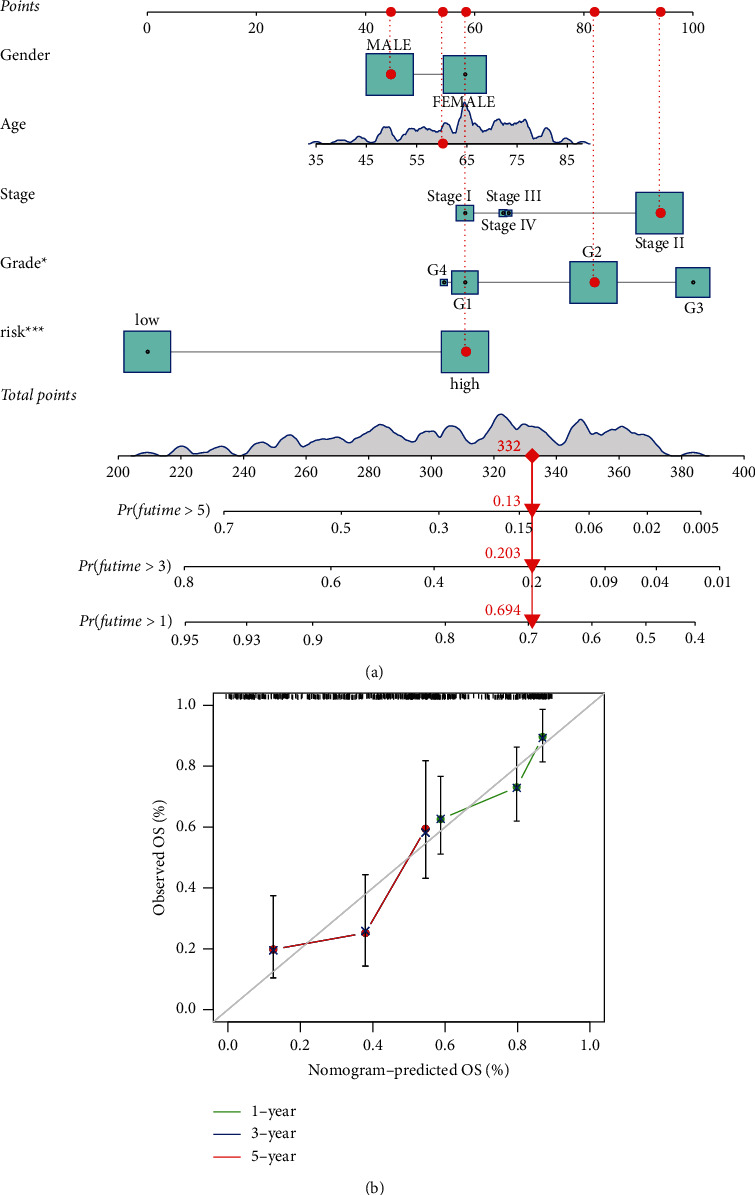
Construction and a nomogram model. (a) A nomogram forecasts the ability of 1-, 3-, and 5-year OS of PC patients. (b) Calibration plot of the nomogram model.

The 1-, 3-, and 5-year OS occurrences were predicted utilizing a nomogram that included risk grade and clinical risk features. In the nomogram, the m5C-LS exhibited superior predictive power when compared to clinical parameters (Figure 8(a)). The observed vs. projected rates of 1-, 3-, and 5-year OS showed perfect consistency in correlation charts (Figure 8(b)).

### 3.6. Evaluation of the Immunotherapy Reaction Based on m5C-LS

**Figure 9:**
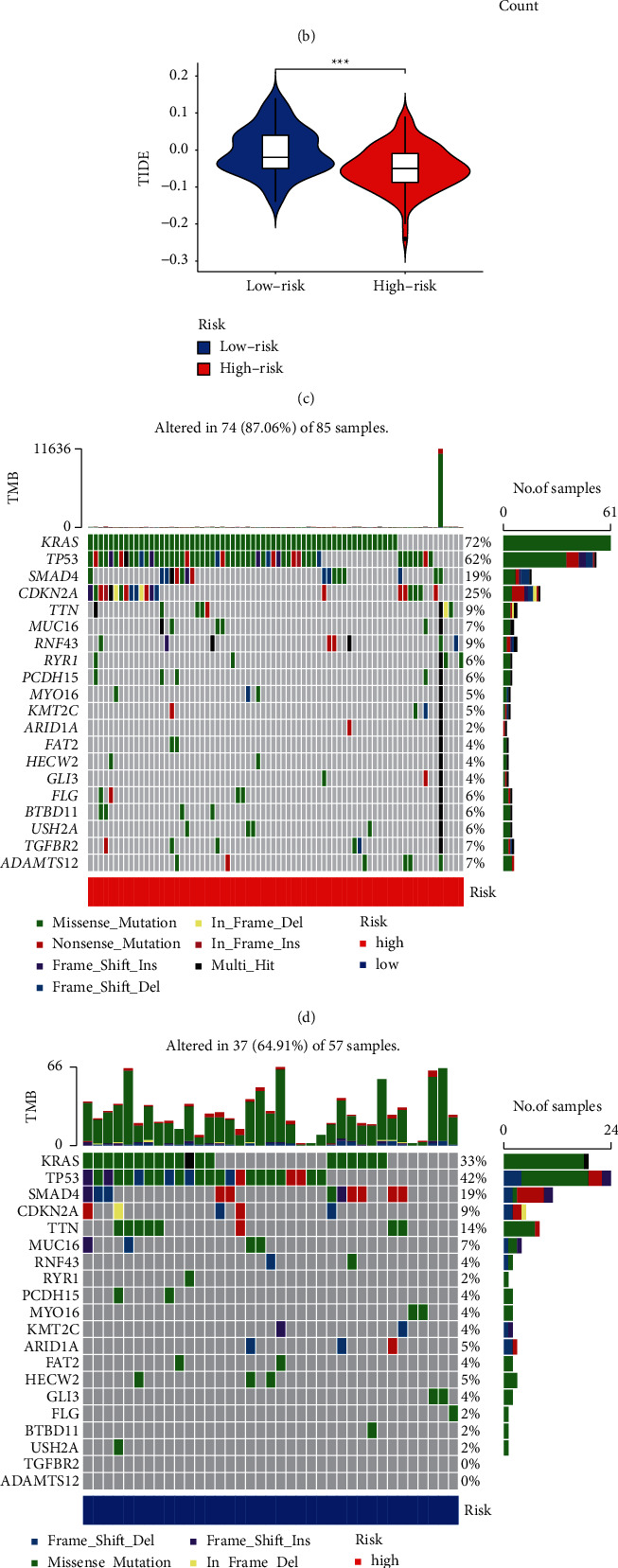
Assessment of the immunotherapy reaction based on the m5C-LS in TCGA entire set. (a) Landscape of immune status between two groups. (b) GO enrichment analysis. (c) Difference of TIDE between two groups. (d, e) Waterfall plot of mutation values in the (d) high-m5C-LS group and (e) low-m5C-LS group. (f) Comparison of TMB between two groups. (g) K-M analysis based on the TMB. (h) K-M analysis combining TMB and the risk signature. ^*∗*^*p* < 0.05, ^*∗∗*^*p* < 0.01, and ^*∗∗∗*^*p* < 0.001.

Based on m5C-LS, the immune status, enrichment pathways, or activities were also investigated in 177 PC samples. The expression of immunological markers differed significantly between the low- and high-m5C-LS groups (Figure 9(a)). We used GO analysis to investigate possible molecular processes of the m5C-LS, which indicated the participation of several immune-related biological processes (Figure 9(b)). The relationship between the m5C-LS and immunotherapy biomarkers was next examined. Predictably, the high-m5C-LS group was more likely than the low-m5C-LS group to react to immunotherapy, suggesting that this m5C-based classifier score might be used to predict the TIDE (Figure 9(c)). The mutation data were evaluated and summarized utilizing R maftools. The variant effect predictor was used to stratify the mutations. Figures 9(d) and 9(e) show the top 20 genes with the largest modifications between two groups. The TMB scores were then generated using TGCA somatic mutation data, and it was discovered that the m5C-LS had a strong connection with TMB (Figure 9(f)). We discovered that a high TMB was linked to a poor OS (*p* = 0.005, Figure 9(g)). We investigated if combining m5C and TMB could become a more stronger prognostic biomarker. We used IGPM and TMB to divide all of the samples into four groups: high TMB/high m5C-LS, low TMB/low m5C-LS, low TMB/high m5C-LS, and low TMB/low m5C-LS. As demonstrated in Figure 9(h), there were significant differences across all groups (*p* < 0.001), with patients in the low TMB/low m5C-LS group having the highest OS. These findings clearly indicated that m5C-LS was connected to tumor aggressiveness.

### 3.7. Discovery of Novel Chemical Compounds Targeting the m5C-LS

We used the pRRophetic algorithm to figure out which drugs might work for PC patients by looking at IC_50_ for each sample in the GDSC database. We found 12 compounds filtering out due to substantial variations in predicted IC_50_ between two groups, with the high-m5C-LS group being more sensitive to the majority of them.  Figure S1 shows the top 12 drugs that could be investigated further in PC.

## 4. Discussion

PC is a highly malignant cancer with a dismal prognosis, and treating it is still tough. Although multimodal therapy including surgery, chemotherapy, radiation, targeted therapy, and immunotherapy has helped patients with PC live longer, the treatment result is still poor [19]. The prognosis and tumor responses of patients with various PC subtypes and clinical features are variable. As a result, for the prognosis and treatment of PC, it is essential to investigate effective and personalized treatments.

Researchers are continuing to describe more molecular properties such as the transcriptome, proteome, and metabolome as high-throughput sequencing methods improve [20]. Chemical alteration of different RNAs at the posttranscriptional level has been shown to control carcinogenesis and tumor growth in recent years. Noncoding RNAs such as microRNA and lncRNA, which have direct functional impacts on gene expression, undergo RNA modification in addition to protein production (mRNA) and effector molecules (tRNA and rRNA) [21]. Among these, N6-methyladenosine (m6A) modification is the most prevalent RNA modification that has participated in the regulation of stem cells [22] and the progression of various cancers. Wang et al. [23] investigated the role of m6A-related lncRNAs in gastric cancer (GC) and developed a predictive signature with a high prognostic value for GC patients. A previous study also discovered that m6A-related genes were significantly associated with malignancy and prognosis in PC [24]. Currently, m5C methylation is another important posttranscriptional modification, which could be catalyzed indicated methyltransferases, mainly including the NOL1/NOP2/SUN (NSUN) family and DNA methyltransferase homologue DNMT [13]. Increasing evidence suggests that m5C methyltransferases have been implicated in many cellular processes and cancer progression. NSUN2, for example, may stabilize the mitotic spindle, promoting tumor cell proliferation, and has been utilized to discover many targets in gallbladder carcinoma, bladder cancer, and a variety of malignancies [25–27]. In breast, ovarian, and prostate cancers, NSUN4 operates as a cancer risk locus [28, 29]. Furthermore, lncRNAs, which are abnormally expressed in distinct cancer cells, play a critical role in cancer-related cellular activities [30]. lncRNAs communicate with DNA, mRNAs, ncRNAs, and proteins to exert their regulatory effects mechanically. However, research into the pathogenic involvement of m5C and lncRNAs in PC development is insufficient, and further research into biological processes and prognostic indicators of PC involving m5C-related lncRNAs is warranted.

To investigate the prognostic function of m5C-related lncRNAs in PC, we firstly selected 243 m5C-related lncRNAs from TCGA dataset, then 13 m5C-related lncRNAs were found to have predictive significance, and three of them were finally used to build a prognostic signature. AC009974.1, one of the three m5C-related lncRNAs, was shown to be implicated in an EMT-related lncRNA signature that predicts prognosis in PC patients [31], indicating that it was a significant prognostic indicator. Abnormal TRAF3IP2-AS1 expression in glioblastoma and renal cell carcinoma has been found in several studies [32, 33], which is likely to be a prognostic marker in tumors. TRPC7-AS1, another lncRNA, was discovered to be overexpressed in hepatoma cells [34]. Following this, we divided PC patients into high- and low-m5C-LS groups based on median scores, with the high-risk group showing worse clinical outcomes. Subgroup studies categorized by gender, age, TNM stage, or tumor stage yielded similar findings. The m5C-LS grouping ability was further confirmed by PCA. Multivariate Cox analysis showed that this model could be an autocephalous risk factor for PC patients' OS. We also created a nomogram that showed perfect consistency between observed and predicted OS rates of 1 year, 3 years, and 5 years. Thus, the established m5C-LS model might lead to the discovery of new biomarkers for future research.

The tumor microenvironment of pancreatic cancer is attracting much attention [35]. We used the TIDE algorithm, a computational technique for modeling tumor immune evasion pathways, to predict cancer treatment by immune checkpoint blockade (ICB) [36]. This result showed that PC patients with high-risk scores had a better response to immunotherapy. TMB refers to the total amount of somatic coding mutations and is linked to the formation of antitumor neoantigens [37]. TMB has been identified as a novel biomarker for predicting PD-L1 therapy response [38]. The TMB in the high-m5C-LS group was greater than in the low-m5C-LS group, implying the immunotherapy in the high-m5C-LS group was more effective. Furthermore, combining TMB with m5C-related lncRNA resulted in an excellent forecast outcome. As a result, this research contributed to our understanding of the molecular biology of m5C-related lncRNAs in PCs.

However, there are still several limitations in this study. First, the data used in the study came from TCGA database, but we lacked a patient cohort to validate them. Second, additional functional experiments on the key three lncRNAs in this signature are required to uncover the special mechanism of m5C methyltransferases in the progression of PC. At last, the predictive value of the m5C-LS needs to be evaluated for clinical applications.

## 5. Conclusion

This study could help us better understand the biological function of m5C-regulated lncRNAs and provide insight into PC prognosis. Furthermore, our constructed m5C-regulated lncRNA signature might guide individual immunotherapy for patients with PC.

## Figures and Tables

**Table 1:** Multivariate Cox analysis of 3 m5C-related lncRNAs.

ID	Coef	HR	HR.95L	HR.95H	*p* value
TRPC7-AS1	−0.6005	0.3505	0.1594	0.7711	0.009
TRAF3IP2-AS1	−1.7203	0.061	0.0126	0.2937	<0.001
AC009974.1	−1.2224	0.1136	0.0273	0.4726	0.002

## Data Availability

All datasets used in this work are included in this manuscript. These data are available in TCGA (https://portal.gdc.cancer.gov/) database.

## Supplementary Materials

Supplementary Materials Figure S1: evaluation of chemical compounds of ICD-50 values between high- and low-m5C-LS groups. Table S1: summary of patients' characteristics. Table S2: lncRNAs correlated with prognosis by univariate Cox regression.
