# Supplementary material for: Signature of m5C-Related lncRNA for Prognostic Prediction and Immune Responses in Pancreatic Cancer
Source: J Oncol. 2022 Feb 15;2022:7467797. doi: 10.1155/2022/7467797 (PMC8863480; doi:10.1155/2022/7467797)
Supplement: Supplementary Materials — Figure S1: evaluation of chemical compounds of ICD-50 values between high- and low-m5C-LS groups. Table S1: summary of patients' characteristics. Table S2: lncRNAs correlated with prognosis by univariate Cox regression. [file 7467797.f1.docx]

**Supplemental material**


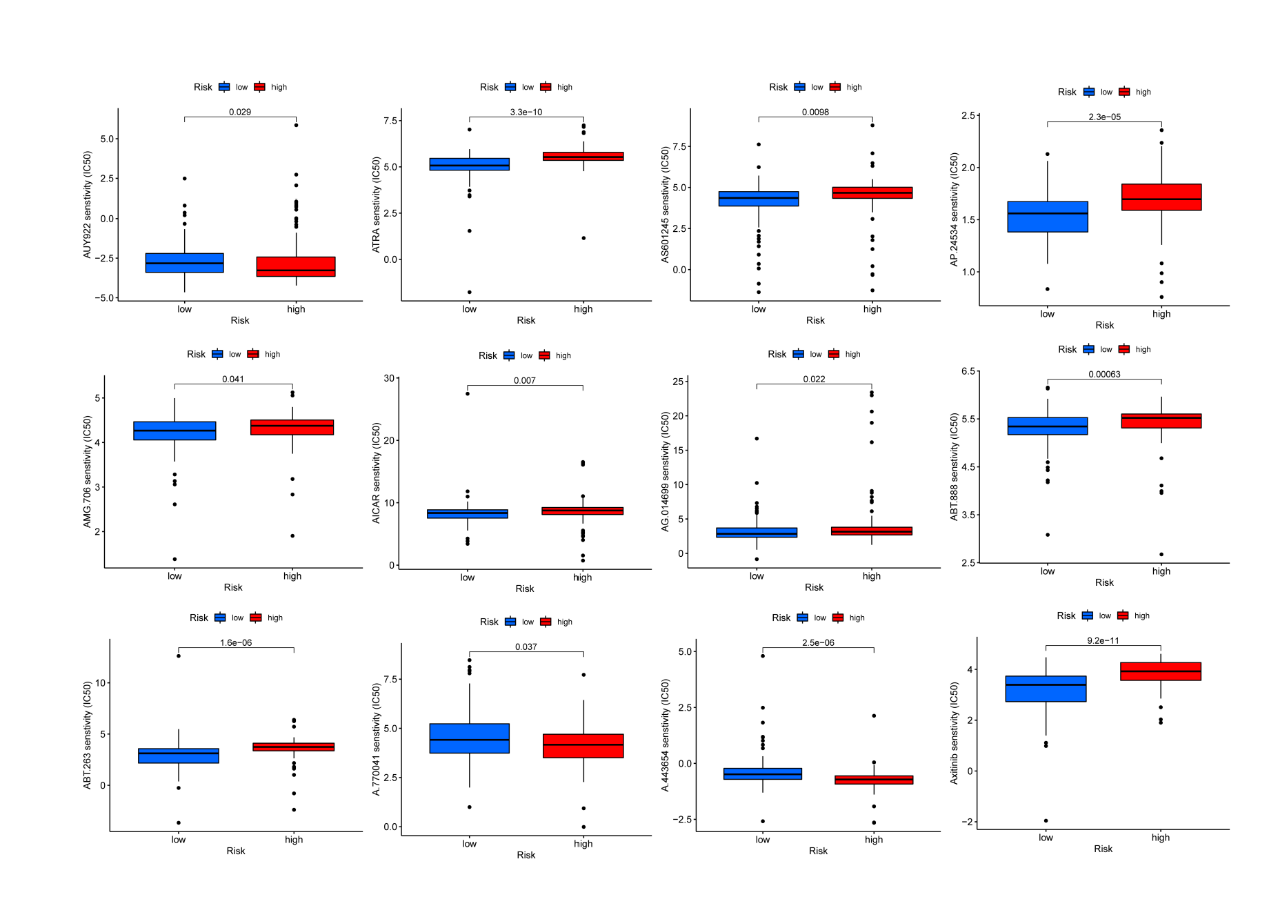


**Figure S1**. **Evaluation of chemical compounds of ICD50 values between high- and low- risk group.**

**Table S1. Summary of patients' characteristics**

| Characteristics |  | Total set(%) | Testing set(%) | Training set(%) | Pvalue |
| --- | --- | --- | --- | --- | --- |
| Age | <=65 | 93(52.54%) | 47(53.41%) | 46(51.69%) | 0.937 |
|  | >65 | 84(47.46%) | 41(46.59%) | 43(48.31%) |  |
| Gender | FEMALE | 80(45.2%) | 42(47.73%) | 38(42.7%) | 0.6021 |
|  | MALE | 97(54.8%) | 46(52.27%) | 51(57.3%) |  |
| Grade | G1 | 31(17.51%) | 16(18.18%) | 15(16.85%) | 0.5414 |
|  | G2 | 94(53.11%) | 46(52.27%) | 48(53.93%) |  |
|  | G3 | 48(27.12%) | 23(26.14%) | 25(28.09%) |  |
|  | G4 | 2(1.13%) | 2(2.27%) | 0(0%) |  |
|  | unknow | 2(1.13%) | 1(1.14%) | 1(1.12%) |  |
| Stage | Stage I | 21(11.86%) | 11(12.5%) | 10(11.24%) | 0.394 |
|  | Stage II | 146(82.49%) | 72(81.82%) | 74(83.15%) |  |
|  | Stage III | 3(1.69%) | 0(0%) | 3(3.37%) |  |
|  | Stage IV | 4(2.26%) | 2(2.27%) | 2(2.25%) |  |
|  | unknow | 3(1.69%) | 3(3.41%) | 0(0%) |  |
| T | T1 | 7(3.95%) | 3(3.41%) | 4(4.49%) | 0.2684 |
|  | T2 | 24(13.56%) | 10(11.36%) | 14(15.73%) |  |
|  | T3 | 141(79.66%) | 73(82.95%) | 68(76.4%) |  |
|  | T4 | 3(1.69%) | 0(0%) | 3(3.37%) |  |
|  | unknow | 2(1.13%) | 2(2.27%) | 0(0%) |  |
| M | M0 | 79(44.63%) | 42(47.73%) | 37(41.57%) | 1 |
|  | M1 | 4(2.26%) | 2(2.27%) | 2(2.25%) |  |
|  | unknow | 94(53.11%) | 44(50%) | 50(56.18%) |  |
| N | N0 | 49(27.68%) | 29(32.95%) | 20(22.47%) | 0.2094 |
|  | N1 | 123(69.49%) | 58(65.91%) | 65(73.03%) |  |
|  | unknow | 5(2.82%) | 1(1.14%) | 4(4.49%) |  |

**Table S2. lncRNAs correlated with prognosis after univariate Cox regression.**

| Symbol | HR | HR.95L | HR.95H | P value |
| --- | --- | --- | --- | --- |
| AL390208.1 | 0.189008 | 0.053092 | 0.672866 | 0.010125 |
| AC005332.6 | 0.416156 | 0.197627 | 0.876328 | 0.021031 |
| AC087501.4 | 0.111321 | 0.013267 | 0.9341 | 0.043096 |
| AC007786.1 | 0.051355 | 0.003695 | 0.713824 | 0.027035 |
| MEG9 | 0.365428 | 0.136605 | 0.977542 | 0.044941 |
| AC010175.1 | 0.291094 | 0.087599 | 0.967312 | 0.043988 |
| AC099329.2 | 2.800205 | 1.030363 | 7.610084 | 0.043529 |
| TRPC7-AS1 | 0.350554 | 0.159492 | 0.770498 | 0.009085 |
| LINC00847 | 0.488853 | 0.266604 | 0.896376 | 0.02069 |
| SUGT1P4-STRA6LP | 0.031564 | 0.002294 | 0.434316 | 0.009782 |
| AL117382.1 | 0.421286 | 0.207372 | 0.855862 | 0.016832 |
| LINC00852 | 0.175677 | 0.034858 | 0.885376 | 0.035075 |
| TRAF3IP2-AS1 | 0.061039 | 0.012683 | 0.293762 | 0.000487 |
| TMEM254-AS1 | 0.286548 | 0.084794 | 0.968339 | 0.044246 |
| ZNF236-DT | 0.243199 | 0.070922 | 0.833951 | 0.024527 |
| AP000894.4 | 0.505949 | 0.284348 | 0.90025 | 0.020483 |
| AF111169.3 | 0.272511 | 0.084477 | 0.87909 | 0.029584 |
| ZBED3-AS1 | 0.027701 | 0.001634 | 0.469696 | 0.013021 |
| AC005062.1 | 0.10624 | 0.019743 | 0.571696 | 0.009023 |
| CH17-340M24.3 | 0.396168 | 0.225419 | 0.696254 | 0.001289 |
| KDM7A-DT | 0.544725 | 0.318997 | 0.930184 | 0.026078 |
| ST3GAL5-AS1 | 0.134712 | 0.023125 | 0.784763 | 0.025779 |
| AC010615.2 | 0.070551 | 0.007027 | 0.708331 | 0.02426 |
| LINC01091 | 0.167441 | 0.037311 | 0.751424 | 0.019646 |
| PAN3-AS1 | 0.31772 | 0.129692 | 0.778352 | 0.012139 |
| LINC02600 | 0.364224 | 0.138917 | 0.954949 | 0.040005 |
| CASC8 | 1.632594 | 1.109402 | 2.402523 | 0.012895 |
| APTR | 0.502365 | 0.266622 | 0.946548 | 0.033178 |
| LINC00857 | 1.663568 | 1.092194 | 2.533853 | 0.017752 |
| AC021242.3 | 0.030491 | 0.001175 | 0.791178 | 0.035645 |
| AC092171.5 | 0.578511 | 0.340073 | 0.984128 | 0.043489 |
| AC002059.1 | 0.397382 | 0.167894 | 0.94055 | 0.035782 |
| AC067817.2 | 0.331738 | 0.113386 | 0.970575 | 0.043958 |
| AC090948.3 | 0.348759 | 0.136796 | 0.889156 | 0.027384 |
| AC090114.2 | 0.343101 | 0.144319 | 0.815682 | 0.015475 |
| LINC02004 | 3.103666 | 1.309834 | 7.354171 | 0.010077 |
| AC002401.4 | 1.747871 | 1.164985 | 2.622398 | 0.006982 |
| AC027575.2 | 0.415529 | 0.204394 | 0.84476 | 0.015266 |
| FAM27E3 | 0.206154 | 0.047257 | 0.899328 | 0.035628 |
| AL117335.1 | 0.201083 | 0.041555 | 0.973033 | 0.046158 |
| AC017048.3 | 3.702622 | 1.071036 | 12.80013 | 0.038602 |
| AC009974.1 | 0.113645 | 0.027323 | 0.472692 | 0.002787 |
| AC022098.1 | 0.1936 | 0.040222 | 0.93185 | 0.04056 |
| AL031775.1 | 0.187781 | 0.052421 | 0.67266 | 0.010198 |
| TMEM105 | 2.085628 | 1.103541 | 3.941714 | 0.023616 |
